# COVID-19 and Influenza Co-infection: A Systematic Review and Meta-Analysis

**DOI:** 10.3389/fmed.2021.681469

**Published:** 2021-06-25

**Authors:** Masoud Dadashi, Saeedeh Khaleghnejad, Parisa Abedi Elkhichi, Mehdi Goudarzi, Hossein Goudarzi, Afsoon Taghavi, Maryam Vaezjalali, Bahareh Hajikhani

**Affiliations:** ^1^Department of Microbiology, School of Medicine, Alborz University of Medical Sciences, Karaj, Iran; ^2^Non Communicable Diseases Research Center, Alborz University of Medical Sciences, Karaj, Iran; ^3^Department of Microbiology, School of Medicine, Shahid Beheshti University of Medical Sciences, Tehran, Iran; ^4^Medical Microbiology Research Center, Qazvin University of Medical Sciences, Qazvin, Iran; ^5^Department of Pathology, School of Medicine, Shahid Beheshti University of Medical Sciences, Tehran, Iran

**Keywords:** coronavirus, COVID-19, influenza virus, co-infection, meta-analysis, systematic review

## Abstract

**Background and Aim:** Co-infection of COVID-19 with other respiratory pathogens which may complicate the diagnosis, treatment, and prognosis of COVID-19 emerge new concern. The overlap of COVID-19 and influenza, as two epidemics at the same time can occur in the cold months of the year. The aim of current study was to evaluate the rate of such co-infection as a systematic review and meta-analysis.

**Methods:** A systematic literature search was performed on September 28, 2019 for original research articles published in Medline, Web of Science, and Embase databases from December 2019 to September 2020 using relevant keywords. Patients of all ages with simultaneous COVID-19 and influenza were included. Statistical analysis was performed using STATA 14 software.

**Results:** Eleven prevalence studies with total of 3,070 patients with COVID-19, and 79 patients with concurrent COVID-19 and influenza were selected for final evaluation. The prevalence of influenza infection was 0.8% in patients with confirmed COVID-19. The frequency of influenza virus co-infection among patients with COVID-19 was 4.5% in Asia and 0.4% in the America. Four prevalence studies reported the sex of patients, which were 30 men and 31 women. Prevalence of co-infection with influenza in men and women with COVID-19 was 5.3 and 9.1%, respectively. Eight case reports and 7 case series with a total of 123 patients with COVID-19 were selected, 29 of them (16 men, 13 women) with mean age of 48 years had concurrent infection with influenza viruses A/B. Fever, cough, and shortness of breath were the most common clinical manifestations. Two of 29 patients died (6.9%), and 17 out of 29 patients recovered (58.6%). Oseltamivir and hydroxychloroquine were the most widely used drugs used for 41.4, and 31% of patients, respectively.

**Conclusion:** Although a low proportion of COVID-19 patients have influenza co-infection, however, the importance of such co-infection, especially in high-risk individuals and the elderly, cannot be ignored. We were unable to report the exact rate of simultaneous influenza in COVID-19 patients worldwide due to a lack of data from several countries. Obviously, more studies are needed to evaluate the exact effect of the COVID-19 and influenza co-infection in clinical outcomes.

## Introduction

The severe acute respiratory syndrome coronavirus 2 (SARS-CoV-2) causes a rapid spreading viral pneumonia; known as coronavirus disease 2019 (COVID-19) originated from China in December 2019, and now become a worldwide public health emergency and finally becomes a global pandemic (1).

Recognition of COVID-19 is critical as it allows effective infection control measures and potentially beneficial antiviral therapy to be introduced, but the risk of COVID-19 co-infection should not be ignored.

COVID-19 co-infections with other respiratory pathogens which may complicate the diagnosis, treatment, prognosis of COVID-19 emerge new concern. These co-infections may even increase the disease symptom and mortality rate (2). The damage of respiratory ciliated cells due to some viral infections can also facilitate the conditions for an infection with SARS-CoV-2. The overlap of COVID-19 and influenza, as two epidemics at the same time can occur in the cold months of the year as seasonal influenza period begin. Both influenza virus A/B and SARS-CoV-2 are transmitted via close contact, respiratory droplets and contaminated surfaces, and cause a wide range of asymptomatic or mild to severe disease such as flu-like symptoms, pneumonia, loose of taste and smell, and even death (3–5).

There have been several studies published from different parts of world about COVID-19 co-infection with other pathogens especially influenza virus A/B. However, a systematic review that summarizes the results of existing studies has not yet been conducted.

Due to the importance of this issue, the aim of the present study was to systematically review the literature on COVID-19 co-infections with the influenza virus (type A and/or B) and potentially conduct a meta-analysis if possible.

## Methods

The present systematic review and meta-analysis was conducted according to the “Preferred Reporting Items for Systematic Reviews and Meta-Analyses” (PRISMA) (6).

### Search Strategy and Study Selection

The three most important electronic databases included PubMed, Web of Science, and Embase were searched on September 28, 2019 to identify studies published in English from December 2019 to September 2020 as follows: (“COVID-19”[Title/Abstract] OR “novel coronavirus 2019”[Title/Abstract] OR “2019 ncov”[Title/Abstract] OR “nCoV”[Title/Abstract] OR “severe acute respiratory syndrome coronavirus 2”[Title/Abstract] OR “SARSCoV-2”[Title/Abstract]) AND (“influenza”[Title/Abstract] OR “orthomyxoviridae”[MeSH Terms] OR “influenza virus”[Title/Abstract] OR “flu”[Title/Abstract] OR “flu”[Title/Abstract]).

In addition, the reference lists of covered studies were also checked to avoid missing suitable publications. Two different investigators independently checked this process (MG, AT). PICO algorithm was adopted to define inclusion and exclusion criteria for study selection. Accordingly, we evaluated the data on P (Patient, Population or Problem) = patients with COVID-19, I (Intervention or exposure) = influenza virus infection, C (Comparison) = not available, and O (Outcome) = co-infection COVID-19 and influenza A/B.

All clinical studies investigating the presence of influenza virus infection in patients with COVID-19 were selected, except articles that reported only the prevalence of COVID-19 or the prevalence of influenza alone, review articles, abstracts presented in conferences, and duplicate studies. Relevant prevalence studies, case reports and case series were included. In the next step, titles and abstracts of all selected papers were screened by two investigators (SKN, PA).

### Data Extraction

First author name; year of publication; type of study, country where the study was conducted; age and gender of patients, number of patients with confirmed COVID−19, number of patients with influenza co-infection and type of influenza virus were extracted from all eligible articles, and transfer to a data extraction form. To avoid any bias, two authors independently recorded the data (SKN, PA). The discrepancy was resolved by discussing between authors (BH, MD, and MG).

### Quality Assessment

Quality assessment of included publications was performed using a checklist provided by the Joanna Briggs Institute (JBI) which assist in assessing the trustworthiness, relevance and results of published papers (7). Two investigators (HG, MV) checked the quality of included studies andthe publication bias independently.

### Statistical Analysis

Statistical analyses were performed with STATA (version 14, IC; Stata Corporation, College Station, TX, USA). Meta-analysis of the continent-, country-, age-, sex-, and influenza virus type- specific prevalence rates of co-infection observed in the considered studies were conducted. We estimated the pooled proportion of co-infected patients. The pooled frequency with 95% confidence intervals (CI) was assessed using the fixed-effects (FEM), and the random effects models (REM). We assessed statistical heterogeneity using the I^2^ statistical method. Cochran's Q and the I2 statistic were used to determine between-study heterogeneity. Begg's and Egger's tests were used to measure publication bias statistically (*p* < 0.05 was considered statistically relevant publication bias).

## Results

**Figure 1:**
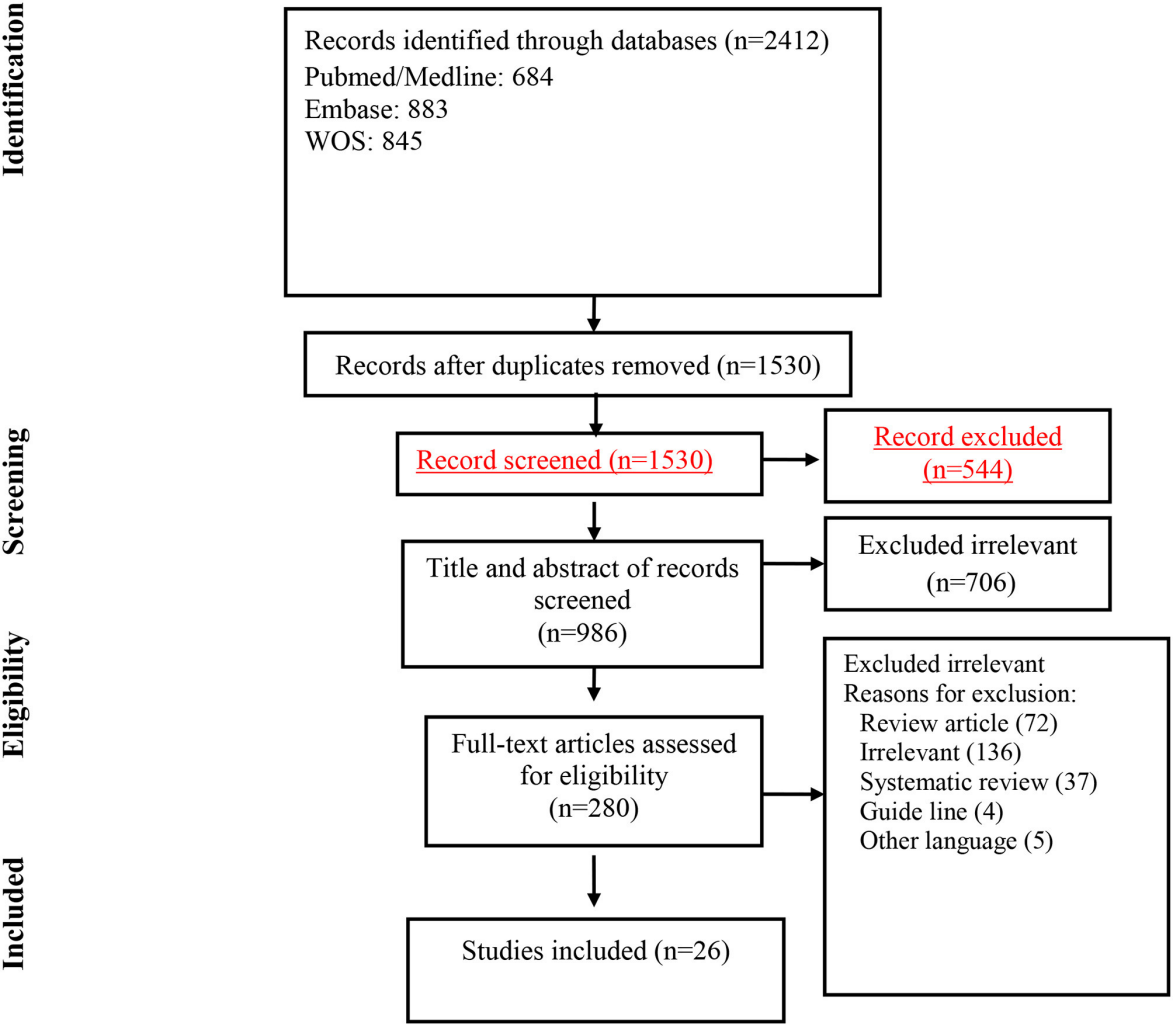
Flow chart of study selection for inclusion in the systematic review and meta-analysis.

Our initial search retrieved 2,412 articles, after removing duplicates, 1,530 articles remain for the secondary screening. Following title and abstract screening 1,250 of the chosen articles were excluded. Guidelines, review articles, duplicate articles, systematic reviews, unrelated articles, and articles in languages other than English were among the excluded articles. After reviewing the full text of 280 studies, eventually, 26 studies met the inclusion criteria and were included for the final analysis (Figure 1). Final selected articles encompass 11 prevalence studies, 15 case report/case series. Tables 1, 2 summarized the characteristics of the included articles.

**Table 1:** Characteristics of included prevalence studies.

**First author**	**Published time**	**Country**	**Patients with COVID-19**	**Patients with COVID-19–Influenza co-infection (%)**	**IV-A**	**IV-B**	**Co-infected patients**	
							**Mean age**	**Male/Female**
Castillo et al. (8)	July, 2020	USA	42	1 (2.4)	1	0	21	1/0
Ding et al. (9)	March, 2020	China	115	5 (4.3)	3	2	50.2	2/3
Garazzino et al. (10)	May, 2020	Italy	168	1 (0.6)	1	nr	nr	Nr
Hashemi1 et al. (11)	July, 2020	Iran	105	23 (21.9)	23	0	nr	14/9
Hu et al. (12)	March, 2020	China	70	32 (45.7)	32	0	62.8	13/19
Kim et al. (13)	April, 2020	USA	116	1 (0.9)	1	0	74	Nr
Leuzinger et al. (14)	July, 2020	Switzerland	930	2 (0.2)	2	0	>16	Nr
de Suoza Luca et al. (15)	May, 2020	Brazil	115	1 (0.9)	0	1	36	Nr
Ma et al. (16)	Jun, 2020	China	250	3 (1.2)	2	1	nr	Nr
Takahashi et al. (17)	Sep, 2020	USA	902	3 (0.3)	nr	Nr	nr	Nr
Zhu et al. (18)	May, 2020	China	257	7 (2.7)	2	5	15–44	Nr

*All studies used real time-polymerase chain reaction (RT-PCR) as their detection method except Hu study which used Elisa method, Ding et al., and Hashemi et al. which did not provided information of their detection method. nr, not reported; IV-A, Influenza Virus A; IV-B, Influenza Virus B*.

**Table 2:** Characteristics of included case report/case series.

**First author**	**Type of study**	**Published time**	**Country**	**Patients with COVID-19**	**Patients with COVID-19 Influenza co-infection**	**IV-A**	**IV-B**	**Co-infected patients**	
								**Mean age**	**Male/Female**
Azekawa et al. (19)	Case report	April, 2020	Japan	1	1	1	0	78	0/1
Benkovic et al. (20)	Case report	Oct, 2020	USA	4	1	1	0	65	1/0
Cuadrado-Payán et al. (21)	Case series	May, 2020	Spain	4	4	3	2	67	3/1
Danis et al. (22)	Case series	Apr, 2020	France	13	1	1	0	9	1/0
Huang et al. (23)	Case report	Jun, 2020	China	1	1	0	1	48	0/1
Kakuya et al. (24)	Case report	May, 2020	Japan	3	1	1	0	11	1/0
Khodamoradi et al. (25)	Case series	April, 2020	Iran	4	4	4	0	57	3/1
Konala et al. (26)	Case series	May, 2020	USA	3	3	3	0	53.3	1/2
Konala et al. (27)	Case report	April, 2020	USA	1	1	1	0	66	0/1
Li et al. (28)	Case report	Jun, 2020	China	1	1	1	0	10	1/0
Miatech et al. (29)	Case series	Oct, 2020	China	81	4	2	2	61.5	2/2
Sang et al. (30)	Case report	May, 2020	USA	1	1	1	0	58	0/1
Singh et al. (31)	Case series	Aug, 2020	USA	3	3	1	2	59.6	1/2
Wu et al. (32)	Case report	June, 2020	China	1	1	1	0	69	1/0
Zou et al. (33)	Case series	Jun, 2020	China	2	2	2	0	7.7	1/1

*All studies used Real time-Polymerase Chain Reaction (RT-PCR) as their detection method. nr, not reported; IV-A, Influenza Virus A; IV-B, Influenza Virus B*.

### Prevalence Studies

Eleven prevalence studies were evaluated in the present review [4 reported from China (36.3%), 3 from USA (27.3%), and Brazil, Switzerland, Italy, and Iran each reported 1 study (9.1%)]. These studies had 3,070 participants with COVID-19 of which 79 patients had influenza co-infection. Sixty seven and 9 patients had an influenza co-infection A or B, respectively. The pooled prevalence of Influenza co-infection among patients with COVID-19 was 0.8 % (95% CI: 0.4–1.3).

**Figure 2:**
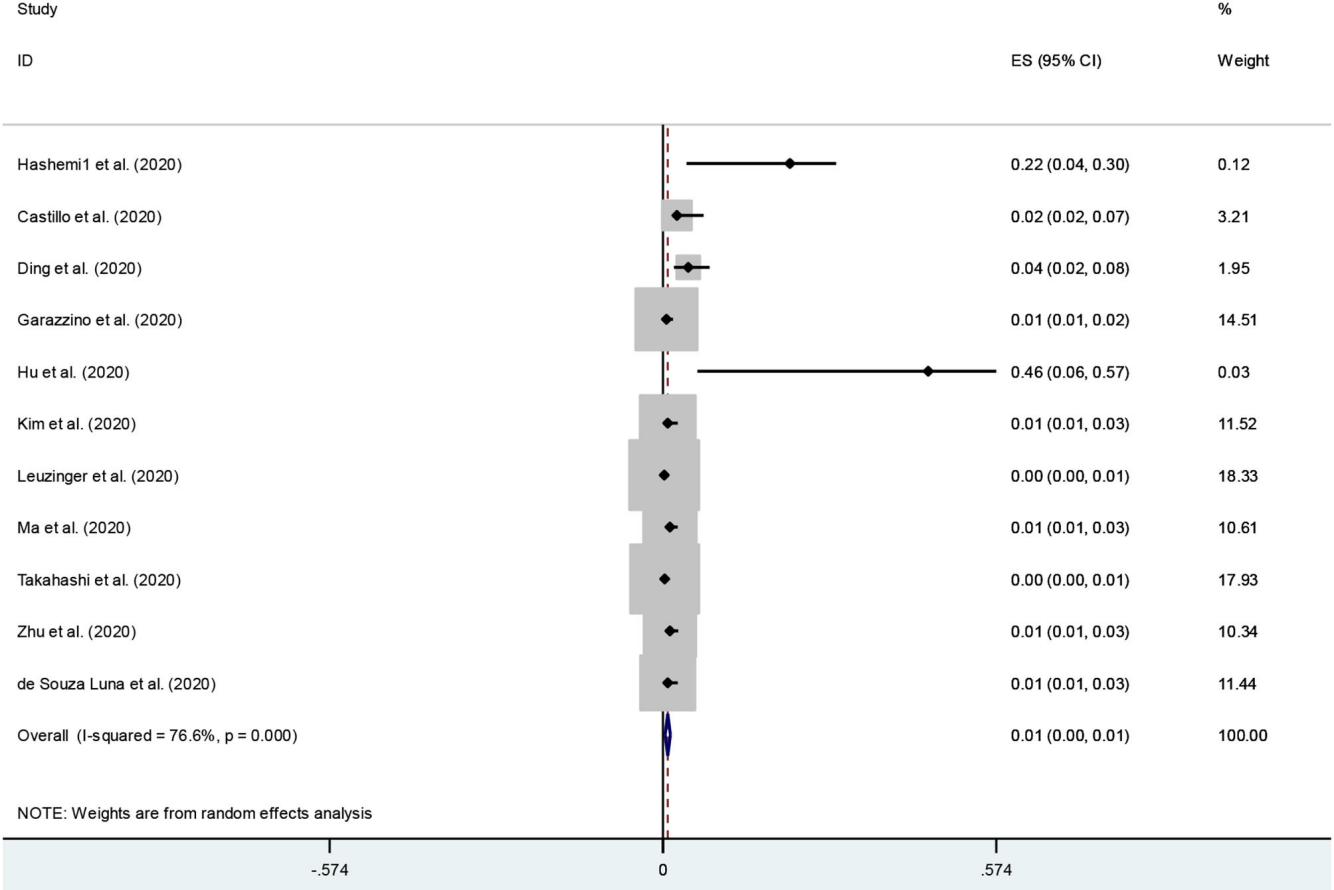
Forest plot of the meta-analysis of data from single studies.

An examination of publication bias was carried out by visual observation of the funnel plot (Supplementary Figure 1). Given the small number of studies, the funnel plots revealed some asymmetry. Then, to provide statistical evidence of funnel plot asymmetry, Egger's and Begg's tests were used. The results showed no signs of publication bias (*p*-values for Egger's and Begg's tests were 0.9 and 0.3, respectively). Forest plot and Galbraith of the meta-analysis on the prevalence of COVID-19 and Influenza co-infection among patients with COVID-19 are shown in Figure 2, and Supplementary Figure 2, respectively.

The meta-analysis of prevalence studies revealed that the frequency of influenza virus co-infection among patients with COVID-19 was 4.5% (95% CI 0.1–7.9) in Asia (5 studies, 70 patients), and 0.4 % (95% CI 0.0–0.7) from the American continent (4 studies, 6 patients). There were no reports of co-infection with influenza virus A/B in patients with COVID-19 from Africa or Oceania at the time of this study. The prevalence of co-infection with influenza in men (30 patients in 4 studies) and women (31 patients in 3 studies) with COVID-19 was 5.3 and 9.1%, respectively. The rate of co-infection in the age groups of <50 years, and more than 50 years was 1.7 and 4.6%, respectively. Table 3 shows more details of subgroup analysis of the studies.

**Table 3:** Frequency of Influenza co-infection among patients with COVID-19 based on different subgroups.

**patients with COVID-19 and Influenza**		**Prevalence% (95% CI)**	**Number of studies**	**Number of patients**	**I-squared**
Overall		0.8 (0.4–1.3)	11	79	76.6%
Continent	America	0.4 (0.0–0.7)	4	6	43.4%
	Asia	4.5 (0.1–7.9)	5	70	84.4%
Country	China	3.1 (0.2–6)	4	47	81.7%
	USA	0.7 (0.0–1.4)	3	5	52.6%
Gender	Male	5.3 (1.3–9.4)	4	30	80.1%
	Female	9.1 (0.6–17.2)	3	31	83.5%
Virus type	Influenza virus A	8 (5.6–10.4)	9	67	62.6%
	Influenza virus B	5.5 (2.8–8.3)	4	9	52.9%
Age	<50 years	1.7 (0.4–3)	3	9	51.2%
	More than 50 years	4.6 (1.4–10.6)	3	38	87.6%

### Case Reports/Case Series Studies

Eight case reports (i.e., total 13 patients) and seven case reports (i.e., total 110 patients) highlighted an influenza co-infection in 8 and 21 COVID-19 patients, respectively. Of these 29 patients (i.e., 13 women and 16 men), influenza type distribution was *A* = 22 (75.9%), *B* = 6 (20.7%) or both = 1 (3.4). The mean age of patients was 48 years. Characteristics of these 15 studies which were not taken into account during the meta-analyses are described in Table 2.

As the details of these studies are listed in Table 4, it can be seen that hypertension (58.6%) and diabetes mellitus (48.3%) were among the most prevalent co-morbidities of co-infected patients. Haemodialysis with the prevalence of 17.2% was the third most common co-morbidity in these patients.

**Table 4:** Comorbidities in coinfected patients in case reports/case series studies among a total of 29 evaluated patients.

**Variables**	**Number of studies**	n^*^ (%)
Atrial fibrillation	1	1 (3.4)
Hyperlipidemia	1	1 (3.4)
Hypertension	7	17 (58.6)
Diabetes mellitus	7	14 (48.3)
Rheumatoid arthritis	1	1 (3.4)
Interstitial lung disease	1	1 (3.4)
Chronic obstructive pulmonary disease	1	1 (3.4)
Haemodialysis	4	5 (17.2)
Hypothyroidism	1	1 (3.4)
Dyslipidemia	2	2 (6.9)
Myocardial infarction	1	1 (3.4)
Gastroesophageal reflux disease	1	1 (3.4)
Chronic kidney disease	3	3 (10.3)
Congestive heart failure	1	1 (3.4)
Coronary artery disease	1	1 (3.4)

* *n, number of co-infected patients with any variables*.

The clinical symptoms were also checked in patients with COVID-19 and influenza virus co-infection. Fever, cough, and shortness of breath were the most common clinical manifestations reported in this group of patients with a frequency of 89.4, 79.3, and 24.1 percent, respectively (Table 5).

**Table 5:** Clinical manifestation of co-infected patients in case reports/case series studies among a total of 29 evaluated patients.

**Variables**	**Number of studies**	n^*^ (%)
Cough	11	23 (79.3)
Fever	14	26 (89.4)
Respiratory distress	1	1 (3.4)
Dyspnoea	3	6 (20.7)
Tachypnoea	2	5 (17.2)
Bronchospasm	1	4 (13.8)
Sputum production	2	2 (6.9)
Gastrointestinal symptoms	2	2 (6.9)
Malaise	2	2 (6.9)
Headache	2	5 (17.2)
Shortness of breath	4	7 (24.1)
Body pain	1	2 (6.9)
Myalgia	5	6 (20.7)

* *n, number of co-infected patients with any variables*.

Based on the results of included studies, nasopharyngeal swabs were the most common method of sampling for molecular evaluations (52%). Throat swab and sputum were only used for two and one patient, respectively.

After reviewing the laboratory findings recorded in the evaluated articles, it was found that the use of nasopharyngeal swabs was the most common method of sampling to diagnose the infection in patients. It was also found that elevated C-reactive protein (CRP) (55.2%), erythrocyte sedimentation rate (ESR) (34.5%), and increased D-dimer (24.1%) were the most commonly reported findings, respectively. Leukopenia (3.4%) was the least reported abnormality.

The outcomes of COVID-19-Influenza co-infection were reported in 11 studies. According to them, 2 of 29 patients died (6.9%), and 17 out of 29 patients recovered (58.6%). Table 6 provides more information in this regard.

**Table 6:** Laboratory and imaging findings of co-infected patients in case reports/case series studies among a total of 29 evaluated patients.

**Variables**	**Number of studies**	n^*^ (%)	
Laboratory finding	Leukopenia	1	1 (3.4)
	Lymphopenia	3	6 (20.7)
	Leukocytosis	2	2 (6.9)
	Elevated blood urea nitrogen	3	3 (10.3)
	Elevated creatinine	3	4 (13.8)
	Elevated interleukin-6 levels	2	6 (20.7)
	Elevated ESR	4	10 (34.5)
	Elevated creatine kinase	1	3 (10.3)
	Elevated Lactate dehydrogenase	3	5 (17.2)
	Elevated C-reactive protein	5	16 (55.2)
	Elevated D-dimer	3	7 (24.1)
Imaging	Chest X-ray: lung infiltrates	10	19 (65.5)
	CT Scan: ground-glass opacities	6	9 (31.0)

* *n, number of co-infected patients with any variables; ESR, erythrocyte sedimentation rate; CT, computerized tomography*.

In the case reports/case series studies, the drugs used for treatment of patients with COVID-19 co-infected with influenza virus divided into sections such as antiviral drugs, antibacterial drugs, and a combination of drugs (Table 5). Oseltamivir was the most widely used antiviral drugs reported in 8 studies. Hydroxychloroquine was also one of the most widely used drugs, as reported by 4 studies, 31% of evaluated patients received this agent. Among the antibacterial drugs azithromycin and ceftriaxone were more common antibiotics each was used in 4, and 3 studies, respectively. Details of the treatment regimens used in the reviewed studies are presented in Table 7.

**Table 7:** Agents used in the treatment of patients with COVID-19 and Influenza co-infection among a total of 29 evaluated patients.

	**Agent**	**Number of studies**	n^*^ (%)	
Antiviral drug	Peramivir	1	1 (3.4)
	Cefaclor	1	1 (3.4)
	Oseltamivir	8	12 (41.4)
	Lopinavir–ritonavir	1	4 (13.8)
	Lianhua qingwen	1	1 (3.4)
Antibacterial drug	Azithromycin	4	7 (24.1)
	Ceftriaxon	3	5 (17.2)
	Levofloxacin	2	3 (10.3)
Other	Hydroxychloroquine	4	9 (31.0)
Combination therapy	Oseltamivir, Levofloxacin	1	1 (3.4)
	Oseltamivir, Cefaclor	1	1 (3.4)
	Oseltamivir, Lianhua qingwen	1	1 (3.4)
	Oseltamivir, Ceftriaxon, Azithromycin	1	1 (3.4)
	Oseltamivir, Hydroxychloroquine, Ceftriaxon, Azithromycin	2	3 (10.3)
	Hydroxychloroquine, Lopinavir–ritonavir	1	4 (13.8)

* *n, number of co-infected patients' receiving each medication*.

## Discussion

COVID-19 as a highly transmissible viral infection which led to thousands of deaths worldwide has challenged the world's healthcare systems (34). Therefore, several studies have been performed to identify how the infection occurs, its symptoms and complications, as well as the factors involved in increasing its severity and mortality, and it is still the subject of studies. Co-occurrence of infections can be one of the causes of exacerbation of this disease. Lansbury et al. in their recent meta-analysis of 30 studies reports the pooled proportions of bacterial and viral co-infections in patients with COVID-19 were 7 and 3%, respectively (35).

In some viral infections, simultaneous infections with other bacterial and viral agents can exacerbate complications and increase disease mortality. The same can be said for SARS-CoV2 infection; however, there is not enough evidence to suggest that such concurrent infections increase disease morbidity or mortality.

COVID-19 often presents with nonspecific flu like respiratory symptoms. Influenza virus infection is thought to be similar to COVID-19 in clinical presentation, transmission mechanism, and seasonal coincidence. Simultaneous infection with influenza and SARS-CoV2 can interfere with the diagnosis and treatment of patients. In addition, this co-infection, especially in high-risk groups of patients, may aggravate the symptoms and complications of the disease (35).

SARS-CoV-2 and influenza virus are both airborne pathogens that affect the respiratory tract. Furthermore, SARS-CoV-2 appears to preferentially infect alveolar type II cells (AT2 pneumocytes), which are also the primary site of influenza virus replication. This can exacerbate the side effects of COVID-19 if there is a concomitant flu infection (36, 37). As a result, the COVID-19 pandemic and seasonal influenza could put a large population at risk of contracting both viruses at the same time. To confirm the role of seasonal flu in the severity of the COVID-19 pandemic, Bai et al. in their research provide the first experimental evidence and reported that the preinfection with influenza virus strongly promotes SARS-CoV-2 virus entry and infectivity in cells and animals. They demonstrated that among the viruses tested; only IAV enhanced SARS-CoV-2 infection (38). This underscores the importance of the risk of influenza infection in patients with COVID-19. Especially in people with underlying factors, the occurrence of such a concomitant infection can aggravate the complications caused by COVID-19.

Due to the onset of influenza infection in the cold seasons of the year, scattered reports have been published about the influenza co-infection in patients with COVID-19 from the countries of the Northern Hemisphere that were in the cold seasons at the time of performing this study. Since there are no accurate statistics on the rate of such concurrent infections, herein, we evaluated the results of prevalence studies as well as case reports and case series in this field in the form of meta-analysis and systematic review.

According to a study recently published from China, concurrent infection of SARS-CoV-2 and influenza virus was common during the initial COVID-19 outbreak in Wuhan, and patients who had co-infection encounter a higher risk of poor health outcomes (39).

Our meta-analysis indicated that overall 1.2% of COVID-19 patients had influenza co-infection. Reports of the frequency of co-infection between COVID-19 and influenza vary from different parts of the world. Lansbury et al. estimated that 3% of patients hospitalized with COVID-19 were also co-infected with another respiratory virus. Based on their analysis, respiratory syncytial virus and influenza-A being the most common viral pathogens in co-infected patients (35).

It should be noted that all studies reviewed in the present study used the molecular method to confirm the presence of SARS-CoV-2 as well as influenza viruses, except for one study in which the presence of infection was diagnosed serologically through detection of IgM. Since serological test is not highly specific and may result in overestimation of infections the overall rate of co-infection reported in that study was higher than others (12).

Overall, the rate of co-infection with COVID-19 and influenza has been reported between 0.24 and 44% in the reviewed studied. This dispersion and difference can be due to the study population, the underlying conditions of the patients, the method of confirming the infection as well as the time and place of the investigation in terms of the prevalence of influenza.

Influenza co-infection among patients with COVID-19 was more reported from Asia and China country. Given the high population of Asia, as well as China, it is reasonable to be likely that patients with COVID-19, as well as influenza in the region be more prevalent, other studies have reported similar statistics too (40, 41).

Based on the results of prevalence studies that reported the influenza virus type involved in the development of infection in patients with COVID-19, it was found that the prevalence of type A virus was higher than type B. This was to be expected due to the higher overall prevalence of influenza virus type A than type B (42).

Finally we should mention the limitations of our study. Since there is not enough information from many countries, we were not able to fully demonstrate the prevalence of influenza infection in COVID-19 patients worldwide. Many COVID-19 patients with influenza may not have been hospitalized and most of them could have been treated at home. Also, some articles lacked the necessary information to be added to the present study, and we had to exclude them. In addition, only studies published in English were included, which may have caused important studies to be missed. Finally, the heterogeneity exists among the included publications. Despite the random effects model allows for the presence of heterogeneity, there may still be some controversy about combining study estimates in its presence.

## Conclusion

Although transmission of other respiratory viruses, including influenza, has decreased with the implementation of preventive measures such as social distancing and mask wearing, however, due to the importance of the issue and the possibility of severe complications in a group of high-risk or hospitalized patients influenza vaccination, especially in the elderly, is strongly recommended.

## Data Availability Statement

The raw data supporting the conclusions of this article will be made available by the authors, without undue reservation.

## Author Contributions

MD and BH designed the study. BH and MG conducted the search strategy. SK, PA, AT, and MV performed the data extraction. HG and BH wrote and edited the manuscript. MD carried out the statistical analysis. All authors contributed to the article and approved the submitted version.

## Conflict of Interest

The authors declare that the research was conducted in the absence of any commercial or financial relationships that could be construed as a potential conflict of interest.
