# Supplementary material for: COVID-19 and Influenza Co-infection: A Systematic Review and Meta-Analysis
Source: Front Med (Lausanne). 2021 Jun 25;8:681469. doi: 10.3389/fmed.2021.681469 (PMC8267808; doi:10.3389/fmed.2021.681469)
Supplement: Supplementary file 1 [file Data_Sheet_1.docx]

**COVID-19 and Influenza Co-infection: A Systematic Review and Meta-analysis**

**Supplementary Figures:**

**Figure S1.** Funnel plot of the meta-analysis on the prevalence of COVID-19 and Influenza co-infection among patients with COVID-19. Solid circles represent each study in the meta-analysis. There were only two off-plot studies that were not excluded due to their importance and the acceptability of I^2^ statistics (Hu et al. and Hashemi1 et al.).

**Figure S2.** Galbraith of the meta-analysis on the prevalence of COVID-19 and Influenza co-infection among patients with COVID-19. All studies are between the two outer parallel lines or a 95% confidence interval except 2 studies (Hu et al. and Hashemi1 et al.).
